# Canine Cancer: Strategies in Experimental Therapeutics

**DOI:** 10.3389/fonc.2019.01257

**Published:** 2019-11-15

**Authors:** Douglas H. Thamm

**Affiliations:** ^1^Flint Animal Cancer Center, Colorado State University, Fort Collins, CO, United States; ^2^Cell and Molecular Biology Graduate Program, Colorado State University, Fort Collins, CO, United States; ^3^University of Colorado Cancer Center, Anschutz Medical Campus, Aurora, CO, United States

**Keywords:** dog, tumor, clinical trial, translational, animal model

## Abstract

Cancer is the most common cause of death in adult dogs. Many features of spontaneously developing tumors in pet dogs contribute to their potential utility as a human disease model. These include similar environmental exposures, similar clonal evolution as it applies to important factors such as immune avoidance, a favorable body size for imaging and serial biopsy, and a relatively contracted time course of disease progression, which makes evaluation of temporal endpoints such as progression free or overall survival feasible in a comparatively short time frame. These criteria have been leveraged to evaluate novel local therapies, demonstrate proof of tumor target inhibition or tumor localization, evaluate potential antimetastatic approaches, and assess the efficacy, safety and immune effects of a variety of immune-based therapeutics. Some of these canine proof of concept studies have been instrumental in informing subsequent human clinical trials. This review will cover key aspects of clinical trials in dogs with spontaneous neoplasia, with examples of how these studies have contributed to human cancer therapeutic development.

## Introduction

Common concerns with regard to the clinical applicability of many murine models of human cancer include immune status, significantly reduced clonal heterogeneity, relative tumor burden, tumor location (orthotopic vs. heterotopic), species-specific differences in drug distribution/metabolism, and differences in *in vivo* drug concentrations that are achievable, among others. These contribute to the observation of extremely poor correlation between results of murine studies and early human clinical trials with anticancer agents (1). More predictive animal models are clearly needed.

More than 1 million new cases of cancer are thought to occur in dogs each year in the United States, and in retrospective studies describing canine mortality, cancer is the most common cause of death with an estimated rate of ~30% (2–4). This large cancer burden in dogs indicates a group of spontaneously occurring tumors, many of which are histologically similar to human tumors. Commonly encountered histotypes include non-Hodgkin lymphoma, malignant melanoma, osteosarcoma (OSA), bladder carcinoma, and multiple brain cancer types among others (2, 5). Client-owned dogs with cancer are being increasingly recognized as a resource for preclinical interrogation of the tolerability, pharmacology, pharmacodynamic effects, and potential efficacy of novel anticancer therapies. This model's potential was discussed in a National Academy of Medicine Workshop on Comparative Oncology that occurred in 2015 (http://www.nap.edu/21830) (6).

Clinical trials in client-owned dogs with spontaneous cancer are potentially important translational models, owing to dogs' relative outbreeding, large size, immunocompetence, and physiological/biological similarity to humans. Spontaneous canine tumors naturally develop treatment resistance, as well as spontaneous recurrence and metastasis. Absolute tumor burdens in dogs are more similar to humans, which may be informative with regard to biological factors such as clonal heterogeneity and hypoxia. The comparatively large size of canine tumors (vs. rodent tumors) also allows for serial tissue collection and imaging over time (2, 7). This is due partly to the fact that these patients are commonly sedated or anesthetized for procedures, moderating concerns over patient discomfort. While clinical case management and data collection are of very high quality, the relative cost of veterinary oncology clinical trials are 10–20% of what similar trials in physician-based oncology would be.

Dogs may also be more reliable models for assessing toxicity of novel therapies than are rodents. As in human patients, canine patients are monitored for hematologic and biochemical toxicities via routine clinical pathology, and sophisticated monitoring (e.g., 24 hour continuous electrocardiographic telemetry, continuous blood pressure measurement, ophthalmologic monitoring, echocardiography, gait analysis, advanced imaging) can be performed as-needed. Unlike in laboratory settings, supportive care (e.g., antiemetics, antidiarrheals, antibiotics, etc.) is also used in client-owned animals similarly to its employment in human patients. Universally accepted grading systems for adverse events from antineoplastic therapy (8, 9), as well as universally accepted tumor response criteria (10, 11), are published.

The Comparative Oncology Trials Consortium (COTC: https://ccr.cancer.gov/Comparative-Oncology-Program/sponsors/consortium) is a network of more than 20 academic veterinary oncology centers, centrally managed by the Comparative Oncology Program, housed within the NIH-NCI-Center for Cancer Research. Its central goal is to plan and perform clinical trials in dogs with cancer to evaluated novel potential therapies for human cancer, with the goal of answering biological questions to inform development for future human clinical trials. COTC sponsored trials are usually pharmacokinetically/pharmacodynamically intensive, with the results incorporated into the design of future human studies. The launch of this network has improved the ability of potential sponsors and collaborators to access a national cooperative group for the conduct of proof of concept studies in dogs. Potential sponsors work with COTC management to iteratively develop a clinical protocol to address a specific drug development question/questions, which is then put out to the membership for potential participation. COTC sites have the opportunity to participate or decline based on capacity, specialized equipment/techniques that may be required, and/or competing trials at the institution. Trial conduct is governed by a single memorandum of understanding between the participating sites, which streamlines the contractual process.

These important attributes have allowed the preclinical evaluation of novel cancer therapeutics that fall into several broad categories: (1) Local therapy approaches such as surgery, radiation therapy and locally-delivered drug therapy; (2) Proof of target inhibition and proof of tumor targeting; (3) Studies in the minimal residual disease setting; (4) Immunotherapy studies.

## Local Therapy Approaches

As a result of dogs' comparatively large body size and the relative size of their tumors, tumor-bearing dogs can be a unique and informative model for the evaluation of novel local therapies. Surgical and radiation therapy (RT) related studies can utilize the same techniques and equipment as would be used in human patients, without the need for the significant adaptation or miniaturization which could be required for rodents. As stated above, the comparatively similar size and growth rate of dog tumors results in similarities in important microenvironmental parameters such as oxygenation, pH, and interstitial fluid pressure (12–18), and the large tumor size facilitates serial biopsy and measurement of intratumoral parameters over time. As a result, tumor-bearing dogs have been utilized in translational studies of novel surgical approaches, RT, hyperthermia, and regionally-delivered drug therapy.

### Translational Surgical Studies

National Cancer Institute sponsored work by Withrow et al. in the 1980's pioneered surgical protocols for cortical allografts for limb-salvage in bone sarcoma patients. These procedures were co-developed by veterinary and human surgical oncologists and refined in a large number of dogs with spontaneous OSA, mostly of the distal radius. Effects of neoadjuvant RT and chemotherapy on surgical outcome and allograft incorporation were also assessed (19–21). These observations and subsequent refinements developed in dogs led directly to the use of these approaches in human limb-sparing surgery (22). An observation was made regarding the postoperative development of bacterial osteomyelitis and improved metastasis-free and overall survival times in dogs (23). This was subsequently observed in at least one study of humans with OSA (24). Further study of this observation in a murine syngeneic OSA model suggested NK- and monocyte-mediated angiogenesis inhibition as a putative mechanism of action (25).

### Radiation Therapy

Studies of radiation by Gillette et al. in the 1970's and 1980's in both normal and tumor-bearing dogs established many normal tissue RT dose constraints still in use today in human patients (19, 26–37). More recently, studies in tumor-bearing dogs provided critical proof of concept for accurate dosimetry and conformal avoidance during the development of helical tomotherapy, a slice-by-slice image-guided intensity modulated RT strategy that is now commercially available (38, 39).

### Translational Studies of Hyperthermia and Radiation Therapy

A substantial body of literature documents pioneering NCI-funded work by Dewhirst et al. evaluating the effects of hyperthermia and hyperthermia/RT combinations on the tumor microenvironment in canine tumors, especially soft-tissue sarcomas. As a result of their common subcutaneous location and the relative ease with which procedures such as serial biopsy and interstitial probe placement can be performed, meaningful insights into thermal dosimetry, alterations in tumor perfusion and tumor oxygenation, and predictors of clinical response were identified (36, 40–42).

### Locally/Regionally Delivered Therapeutics

Multiple studies of inhaled/pulmonary delivered therapeutics have evaluated safety and provided preliminary evidence of antitumor efficacy in support of human trials. These include evaluation of inhaled doxorubicin, paclitaxel and gemcitabine for the treatment of measurable primary or metastatic pulmonary tumors (43, 44), and nebulized inhaled interleukin-2 (IL-2) containing liposomes for treatment of pulmonary metastatic OSA (45). In addition to the observed objective antitumor responses, the latter study included serially collected bronchoalveolar lavage (BAL) fluid to characterize the local leukocyte population before and after IL-2 therapy. Post-IL-2 BAL samples contained a more than four-fold increase in lymphocytes, with a shifted CD4:CD8 ratio and increased cytolytic activity *ex vivo* (45).

Various intratumor treatments have been evaluated in tumor-bearing dog models. These include attenuated *Clostridium* spores (46), and various intralesional gene therapy approaches (47–50). In many of these studies, serial biopsy was performed to evaluate and characterize immune infiltrates and/or confirm transgene expression. Several novel intralesional chemotherapy approaches (± other local treatments such as RT or hyperthermia) have likewise been evaluated, demonstrating tolerability and preliminary evidence of efficacy (50–55).

## Proof of Target Inhibition or Proof of Tumor Targeting/Accumulation

Owing again to the relative ease of serial biopsy, as well as comparably favorable pharmacokinetic parameters in dogs such as organ-specific blood flow and hepatic enzyme homologies, canine tumors can serve as very useful translational models for evaluation of pharmacokinetic/pharmacodynamic (PK/PD) relationships, demonstration of target inhibition, and/or demonstration of tumor targeting. In these cases, substantial preliminary *in vitro* work is often necessary to confirm target expression, demonstrate similar drug behavior in canine and human tumor cells, and potentially validate reagents and protocols necessary for PD assessment. Importantly, there are certain situations where molecular targets may be present in canine tumors that are histologically very different from human tumors expressing the same target. Examples include expression of mutant KIT protein in canine mast cell tumors (MCT) with a similar mutation expressed in human gastrointestinal stromal tumors (56), and expression of the V600E BRAF mutation, commonly expressed in human melanomas, in canine bladder cancer (57, 58).

### Proof of Drug Target Inhibition

An early example of successful evaluation of a novel targeted agent in dogs with spontaneous neoplasia involves the preclinical evaluation of the “split kinase” inhibitor SU11654 (toceranib phosphate, Palladia™), in dogs with MCT. SU11654 is a structural analog of the human multi-kinase inhibitor sunitinib (Sutent™) with very similar physicochemical properties and IC50's against their intended targets, which include KIT, VEGFR2 and PDGFR-alpha. After initial *in vitro* studies demonstrating canine MCT growth inhibition, apoptosis induction and inhibition of KIT phosphorylation (59), pilot studies were performed in tumor-bearing dogs demonstrating achievement of likely therapeutic drug concentrations in plasma with good tolerability and evidence of antitumor activity (60). Furthermore, inhibition of KIT activation and downstream signaling was demonstrated in biopsy samples prior to and 8 h following the first dose of drug (61). These data provided critical information in support of the human development of sunitinib, which is now approved by the U.S. Food and Drug Administration (FDA) for human renal cell carcinoma, pancreatic neuroendocrine tumors, and gastrointestinal stromal tumors, and led to the FDA approval of toceranib for the treatment of canine MCT (62).

A similar “next to lead” approach has been taken with the selective inhibitor of nuclear export verdinexor (KPT-335), which was evaluated *in vitro* for activity in canine tumor cells, then in tumor-bearing dogs to provide supporting data for development of the human analog selinexor (KPT-330, Xpovio™) (63), now approved by the FDA for the treatment of human multiple myeloma. Verdinexor is now in clinical development as a canine cancer therapeutic.

Rather than evaluating a structural analog to generate preclinical data in tumor-bearing dogs in support of a human clinical candidate, another recent study evaluated PCI-32765 (ibrutinib, Imbruvica™), a first-in-class inhibitor of the Bruton tyrosine kinase (Btk), in dogs with spontaneous B-cell lymphoma prior to first-in-in-human studies (64). Goals of the study were 2-fold: (1) To validate a PD assay to be used in subsequent human trials; (2) To generate preliminary evidence of efficacy, since reliable murine B-cell lymphoma models demonstrating intact B cell receptor signaling were not available. Btk receptor occupancy following ibrutinib treatment was similar in lymphoma tissue and peripheral blood following treatment, providing support that measurement in blood alone would likely be accurate in humans. Furthermore, major antitumor responses were observed in three of eight dogs treated, providing strong impetus to accelerate human clinical development of ibrutinib. Ibrutinib now has FDA approval in humans for the treatment of certain B cell lymphoma subtypes, chronic lymphocytic leukemia, Waldenstrom's macroglobulinemia and graft-vs.-host disease.

### Proof of Tumor Targeting

Canine tumors have been utilized to confirm tumor-specific targeting and/or tumor accumulation of therapeutics. The inaugural COTC trial evaluated a tumor vasculature targeted adeno-associated virus phage vector targeted to alphaV integrins expressed on tumor endothelium and delivering tumor necrosis factor (TNF), in preparation for human trials. Selective targeting of tumor (vs. normal) vasculature was documented through serial biopsy of tumor and proximate normal tissues after intravenous infusion, and tumor-directed expression of TNF was documented. Furthermore, objective antitumor responses were noted in 2 of 14 dogs (65).

Certain bacteria, especially facultative anaerobes, demonstrate tropism for tumor tissues. VNP20009 is a *Salmonella typhimurium* strain that was attenuated through deletion of the *MsbB* gene, contributing to endotoxin production, and the PurI gene, requiring an exogenous source of purines for survival. These deletions reduce toxicity and further restrict colonization to tumor tissues *in vivo*, while diminishing or eliminating survival in the environment. Intravenous infusion of VNP20009 was evaluated in tumor-bearing dogs for safety and evidence of tumor colonization (66). While blood cultures were uniformly negative 7 days following infusion, the organism was isolated from tumor tissue in 42% of dogs. The objective response rate was 15% (10% complete responses). These data supported an NCI-sponsored clinical trial of VNP20009 in human metastatic melanoma (67). No objective antitumor responses were observed in the human melanoma study, however; this could be due to selection of melanoma as the sole human tumor type for study, or due to differences in either tolerability or host (e.g., immune, vascular) response to the bacterium between dog and human. Strategies for geographically targeted cytotoxic drug delivery via hyperthermia and thermosensitive liposomes have also been investigated in canine soft tissue sarcomas (68).

### Proof of Tumor Drug Accumulation

A recent canine clinical trial of the autophagy modulating agent hydroxychloroquine (HCQ), which was published concurrently with a series of human clinical trials, was the first to document substantial accumulation (~100-fold) of HCQ in tumor tissue when compared with plasma, and to demonstrate that there was no correlation between drug concentrations or changes in autophagy in the two compartments. This suggested that peripheral blood is not a good surrogate for tumor HCQ concentration or autophagy-modulatory activity, and that future clinical trials should aim to identify more accurate surrogates of HCQ activity (69).

Another large COTC trial evaluated a series of three distinct indenoisoquinolone-class topoisomerase I inhibitors in dogs with spontaneous lymphoma. Eighty-four dogs with lymphoma were allocated to receive one of three drugs. Tolerability, pharmacokinetics, target engagement and antitumor effects were evaluated. One of the three drugs, LMP744, demonstrated significantly increased accumulation in tumor tissue vs. the other two drugs, and enhanced antitumor activity was ascribed to this increased tumor accumulation (70). Although LMP744 was not originally selected for further human development, the unexpected positive results of the canine trial encouraged the NCI to evaluate LMP744 in humans (ClinicalTrials.gov identifier NCT03030417). This human trial is currently accruing and thus human safety/efficacy data are not currently available.

## Antimetastatic Efficacy

Another potential advantage of canine clinical cancer research is the relatively compressed time line for tumor progression and the spontaneous development of local recurrence, metastasis, and drug resistance. These characteristics allow surgical adjuvant studies against “microscopic residual disease,” with temporal endpoints such as progression free or overall survival, to be conducted relatively expediently. This may be useful especially for agents designed primarily as antimetastatic therapies. Several candidate human therapies have been investigated in this context in tumor-bearing dogs.

Extensive work by Macewen, Kurzman et al. with the peptidoglycan recognition protein agonist and non-specific immune stimulant liposome muramyl tripeptide phosphatidylethanolamine (L-MTP-PE) was performed in dogs with hemangiosarcoma (HSA) and OSA. Randomized placebo controlled trials demonstrated meaningful delays in metastasis and prolongation of overall survival times when surgery and chemotherapy were combined with L-MTP-PE (71, 72). Furthermore, bronchoalveolar lavage performed before and after L-MTP-PE indicated significant enhancement of activation status and *ex vivo* antitumor cytotoxicity in pulmonary alveolar macrophages (73). This work provided critical proof of principle showing delay of metastasis in OSA, which led directly to the performance of a randomized, placebo-controlled trial of surgery, chemotherapy ± L-MTP-PE in human OSA (74). Subsequently, L-MTP-PE (mifamurtide, Mepact™) was granted regulatory approval by the European Medicines Agency for treatment of human OSA.

Another randomized, multicenter surgical adjuvant study compared standard-of-care therapy with carboplatin to treatment with the novel liposomal cisplatin drug SPI-77 in dogs with appendicular OSA. Despite SPI-77's capacity to deliver five times more cisplatin vs. the maximum tolerated dose of free cisplatin, there were no improvements in progression free survival time or overall survival time when compared with conventionally dosed carboplatin. These results, combined with other factors, contributed significantly to the decision to suspend SPI-77's clinical development (75).

In a recent study, dogs with splenic HSA were treated after splenectomy with a combination of doxorubicin and an epidermal growth factor receptor- and urokinase-targeted *Pseudomonas* exotoxin, referred to as eBAT. These targets appear to be conserved in certain human sarcomas, and thus canine HSA may be a valuable translational model despite the distinct histotype and rareness of its human homolog. In addition to very good tolerability, there was the suggestion of improved outcome when eBAT-treated patients were compared with historical canine patients receiving doxorubicin alone (76). The human development path for eBAT is not currently known.

## Immunotherapy

In addition to the advantages discussed above, a unique advantage of spontaneous canine tumors that has been somewhat overlooked is that these tumors have evolved, by necessity, immune-avoidance strategies that are very similar to those utilized by human cancers. This is in stark contrast to syngeneic murine tumor models, where immune tolerance does not evolve similarly. These immune-avoidance strategies include upregulation of immune-suppressive cytokines such as IL-8, IL-10, and transforming growth factor beta (77–80), cooptation of innate immune-suppressive cells such as regulatory T cells (81–83), myeloid-derived suppressor cells (84–86), and “steady state” macrophages (87–90), and upregulation of immune checkpoint molecules such as PD-L1 and B7x (91–95). Thus, successful cancer immunotherapy in dogs requires overcoming of these conserved immune-avoidance strategies just as is required in humans.

In addition to the approaches mentioned in previous sections, a variety of immunotherapy strategies have been investigated in dogs over decades. These range from passive non-specific immunotherapy approaches to early studies with canine chimeric antigen receptor-engineered T (CAR-T) cells. A partial list of immunotherapy approaches investigated in dogs with cancer is provided in Table 1. An exhaustive discussion of these approaches is beyond the scope of this review; however, this issue contains a dedicated article discussing canine tumor immunology and immunotherapy. Several of the approaches outlined in the Table have led to human clinical trials (74, 130, 131).

**Table 1:** Immunotherapy approaches investigated in canine cancer trials.

**Category**	**Therapy type**	**References**
Passive, nonspecific	BCG/other bacterial products	(46, 66, 96–100)
	L-MTP-PE	(71–73, 101)
	Recombinant cytokines	(45, 101–107)
	Intralesional immuno/gene therapy	(47, 48, 108–110)
Passive, specific	Tumor-targeting antibodies	(111, 112)
	Checkpoint inhibitors	(113)
	Whole cell vaccines	(114–119)
	Gene/peptide vaccines	(120–124)
Active, nonspecific	Activated T cells	(125, 126)
	Oncolytic virotherapy	(127, 128)
Active, specific	CAR-T cells	(129)

## Conclusions and Future Directions

In conclusion, there is great potential for studies in dogs with spontaneous cancer to inform development of novel human therapeutics and diagnostics. In general, these studies have a higher potential for success when there is a focused, *a priori* question that canine studies seek to answer, and a plan for utilization of the data generated is in place prior to study commencement. Additionally, utilizing the strengths of the model, especially vis a vis the ability to repeatedly sample tumor tissue, to generate robust PK/PD related data is value-added. These types of data are perhaps more critical in answering questions regarding why a treatment did not work than in supporting how a treatment did work. Was it an issue of insufficient drug exposure? Was there adequate exposure in plasma but not tumor? Was the target appropriately inhibited despite a lack of antitumor activity?

Additionally, successful implementation of studies in dogs generally requires some amount of preclinical work for validation of target expression, validation of drug activity against the canine analog of the target, and selection/validation of PD endpoints to be implemented in subsequent canine clinical trials. A lack of canine-specific reagents often requires some legwork for the validation of cross-reactive antibodies for these types of applications.

Ongoing foundational work has the potential to significantly expand the molecular underpinnings of canine cancer, and facilitate comparisons with human cancer. A number of 1 year administrative supplements to existing NIH P30 grants were recently approved, with the goals of utilizing next-gen sequencing (whole-exome sequencing, RNASeq) to characterize a variety of canine tumor types for quantification of mutational load, identification of driver mutations, and characterization of potential neoantigens for MHC binding. Furthermore, a series of U01 grants were recently funded by the NIH to explore novel immunotherapy approaches in canine cancer to inform human cancer immunotherapy studies. These studies have the potential to expand understanding of the molecular drivers of canine cancer and uncover novel shared molecular targets and pathways for future study.

Several ongoing large-scale longitudinal studies are taking advantage of dogs' foreshortened life spans to answer a variety of questions about life style, environment, aging and cancer incidence, as well as evaluating novel interventions. The Golden Retriever Lifetime Health Study (www.morrisanimalfoundation.org/golden-retriever-lifetime-study) is following 3,000 US golden retrievers from young adulthood to death, to identify environmental, nutritional, genetic, and lifestyle risk factors for cancer and other diseases in dogs. The University of Washington Dog Aging Project (https://dogagingproject.org) seeks to profile and follow up to 10,000 dogs to determine incidence and risk factors for a variety of age-related diseases, as well as pursuing smaller-scale trials with novel anti-aging (and potentially anti-cancer) interventions. The Vaccination Against Canine Cancer Study (www.vaccs.org) is an 800-dog, randomized, placebo-controlled, prospective, multi-center clinical trial seeking to evaluate the evaluate the ability of a multivalent frameshift vaccine to delay or prevent cancer development in healthy older dogs. These three long-term studies have the potential to shed significant light on genetic, environmental, lifestyle, and immunologic risk factors for cancer that may have significant translatability. The results are eagerly anticipated.

## Author Contributions

The author confirms being the sole contributor of this work and has approved it for publication.

### Conflict of Interest

The author declares that the research was conducted in the absence of any commercial or financial relationships that could be construed as a potential conflict of interest.
